# Synthesis and crystal structure of 1,3-bis­{[*N*,*N*-bis­(2-hy­droxy­eth­yl)amino]­meth­yl}-5-{[(4,6-di­methyl­pyridin-2-yl)amino]­meth­yl}-2,4,6-tri­ethyl­benzene

**DOI:** 10.1107/S2056989022007411

**Published:** 2022-07-26

**Authors:** Manuel Stapf, Ute Schmidt, Wilhelm Seichter, Monika Mazik

**Affiliations:** a Institut für Organische Chemie, Technische Universität Bergakademie Freiberg, Leipziger Str. 29, 09599 Freiberg/Sachsen, Germany; Universidad Nacional Autónoma de México, México

**Keywords:** crystal structure, tripodal mol­ecule, O—H⋯O bonding, intra­molecular hydrogen bond

## Abstract

In the crystal structure of the title compound, C_30_H_50_N_4_O_4_, the two bis­(hy­droxy­eth­yl)amino moieties and the 2,4-di­methyl­pyridinyl­amino unit of the mol­ecule are located on one side of the central benzene ring, while the ethyl substituents are oriented in the opposite direction. The dihedral angle between the planes of the aromatic rings is 73.6 (1)°. The conformation of the mol­ecule is stabilized by intra­molecular O—H⋯O (1.86–2.12 Å) and C—H⋯N (2.40, 2.54 Å) hydrogen bonds. Dimers of inversion-related mol­ecules represent the basic supra­molecular entities of the crystal structure. They are further connected *via* O—H⋯O hydrogen bonding into undulating layers extending parallel to the crystallographic *bc* plane. Inter­layer inter­action is accomplished by weak C—H⋯π contacts.

## Abstract 2

A 1,3,5-tris­ubstituted 2,4,6-tri­alkyl­benzene derivative with bis­(hy­droxy­eth­yl)amino and 2,4-di­methyl­pyridinyl­amino substituents was synthesized and its structure determined by single-crystal X-ray diffraction. The crystal packing is stabilized by intra- and inter­molecular hydrogen bonds and weak C—H⋯π contacts.

## 1. Chemical context

The 1,3,5-tris­ubstituted 2,4,6-tri­alkyl­benzene scaffold has shown to be valuable for the construction of various artificial receptors (Hennrich & Anslyn, 2002 ▸). In the course of our research work, we have successfully used this mol­ecular scaffold in the design of acyclic and macrocyclic receptors for neutral (Mazik, 2009 ▸, 2012 ▸; Lippe & Mazik, 2015 ▸; Lippe *et al.*, 2015 ▸; Amrhein *et al.*, 2016 ▸; Koch *et al.*, 2016 ▸; Amrhein & Mazik, 2021 ▸; Köhler *et al.*, 2020 ▸, 2021 ▸) and ionic substrates (Geffert *et al.*, 2013 ▸; Stapf *et al.*, 2015 ▸; Schulze *et al.*, 2018 ▸). Our studies on the mol­ecular recognition of carbohydrates have shown that the participation of different types of recognition groups in the complexation of the substrate favourably influences the binding process (Stapf *et al.*, 2020*a* ▸,*b* ▸; Kaiser *et al.*, 2019 ▸). Such a combination of two types of recognition units, namely heterocyclic and hy­droxy groups, is realised in the tri­ethyl­benzene-based title compound **1** (see also Stapf *et al.*, 2020*a* ▸). The design of the receptors consisting of the aforementioned recognition units was inspired by the nature of the protein binding sites involved in the inter­actions stabilizing the crystalline protein–carbohydrate complexes (Quiocho, 1989 ▸). For example, 2-amino­pyridine can be considered as a heterocyclic analogue of the asparagine/glutamine primary amide side chain. Furthermore, it should be noted that the formation of intra­molecular inter­actions is also one of the factors influencing the binding properties of a receptor mol­ecule (Rosien *et al.*, 2013 ▸). Intra­molecular inter­actions can also be observed in the crystal structure of **1**. 
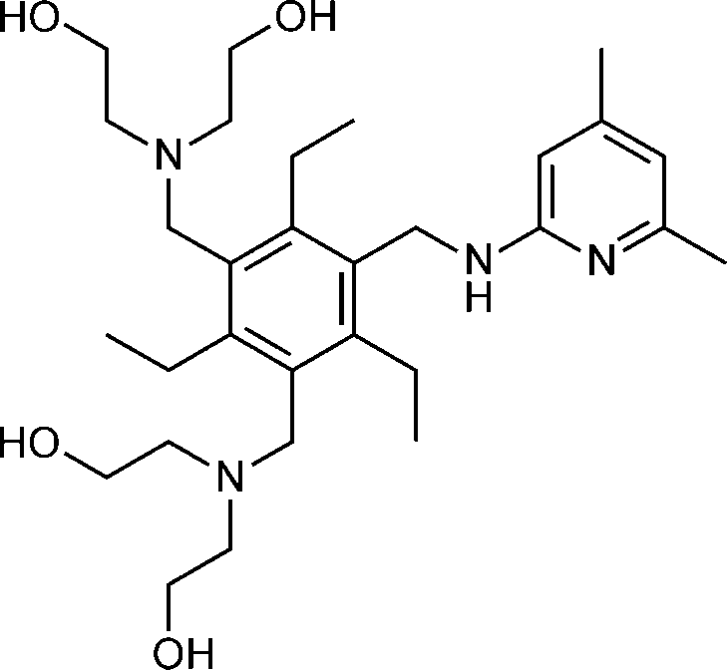


## 2. Structural commentary

**Figure 1:**
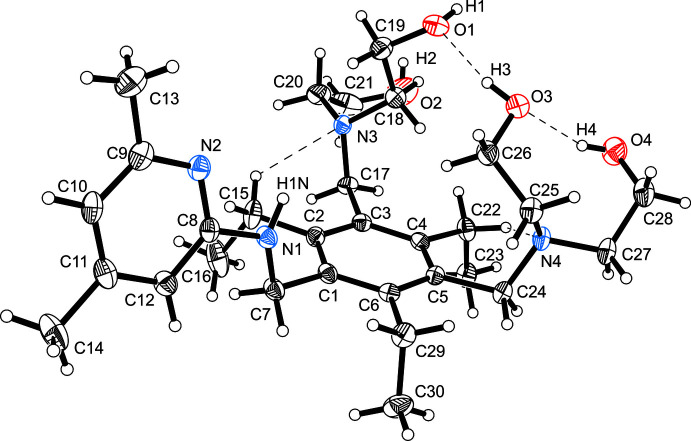
Perspective view of the mol­ecular structure of the title compound including atom numbering. Anisotropic displacement ellipsoids are drawn at a 50% probability level. Dashed lines represent hydrogen-bonding inter­actions. For the sake of clarity, only the major position of the disordered bis­(hy­droxy­eth­yl)amino moiety is shown.

In the title mol­ecule, the structure of which is shown in Fig. 1 ▸, the functionalized side arms are arranged on one side of the central benzene ring, while the ethyl substituents are oriented in the opposite direction. One of the bis­(hy­droxy­eth­yl)amino moieties is disordered over two positions [s.o.f. 0.879 (2)/0.121 (2)]. The inter­planar angle between the aromatic rings is 73.6 (1)°. Within the mol­ecule, three hy­droxy groups create a continuous pattern of O—H⋯O hydrogen bonds [*d*(H⋯O) 1.86–2.12 Å]. The amino nitro­gen atoms N3 and N4 are involved in intra­molecular C—H⋯N hydrogen bonding [*d*(H⋯N) 2.40, 2.54 Å]. The crystal structure contains four potentially solvent-accessible voids with a total volume of 110 Å^3^ per unit cell (Spek, 2015 ▸). The void volume of 27.5 Å^3^ and the maximum residual electron density of 0.55 e Å^−3^ indicate that the voids could be partially occupied by water mol­ecules.

## 3. Supra­molecular features

**Figure 2:**
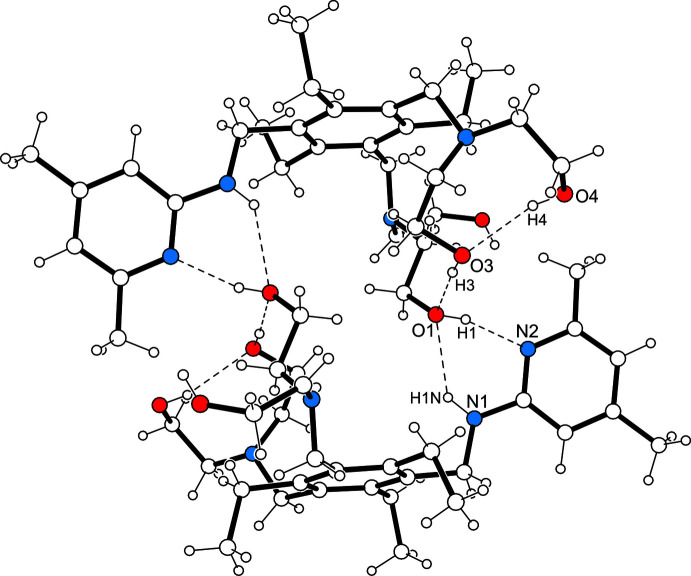
Structure of the mol­ecular dimer including the numbering of atoms involved in hydrogen-bonding inter­actions. For the sake of clarity, only the major position of the disordered hy­droxy­ethyl moiety is shown. Hydrogen bonds are shown as dashed lines.

**Figure 3:**
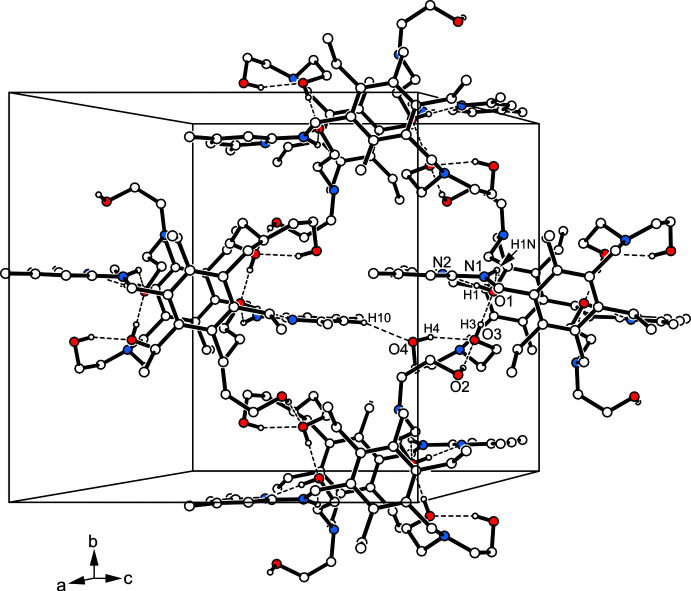
Packing excerpt of the title compound with numbering of coordinating atoms. Oxygen atoms are displayed as red, nitro­gen atoms as blue circles. Hydrogen atoms excluded from inter­molecular inter­actions are omitted for clarity. Hydrogen bonds are shown as broken lines.

**Figure 4:**
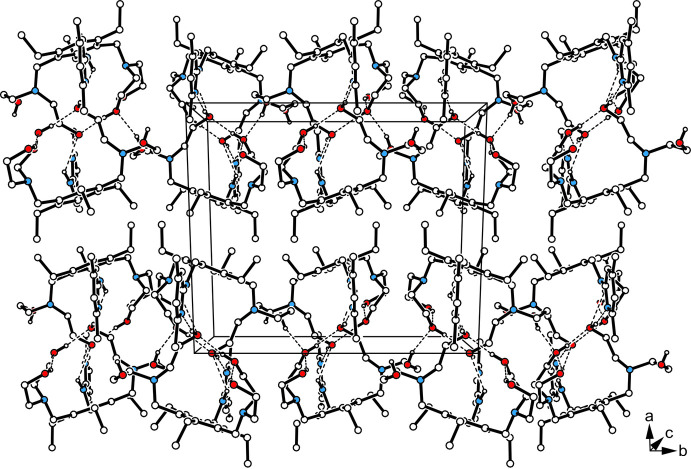
Packing diagram of the title compound viewed down the *c* axis. Oxygen atoms are displayed as red, nitro­gen atoms as blue circles. Hydrogen-bonding inter­actions are shown as dashed lines.

As depicted in Fig. 2 ▸ and Fig. 3 ▸, the crystal structure is constructed of inversion-symmetric mol­ecular dimers held together by O—H⋯N and N—H⋯O hydrogen bonding [*d*(H⋯N) 1.89 (2) Å; *d*(H⋯O) 2.19 (2) Å; graph set 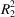 (6) (Etter, 1990 ▸; Bernstein *et al.*, 1995 ▸)]. These dimers are further assembled *via* O—H⋯O [*d*(H⋯O) 1.99 (2) Å] and C—H⋯O [*d*(H⋯O) 2.45 Å] bonds (Desiraju & Steiner, 1999 ▸) into layers extending parallel to the crystallographic *bc* plane (Fig. 4 ▸). As the layer surfaces are defined by the ethyl groups of the mol­ecules, inter­layer association is restricted to weak C—H⋯π contacts (Nishio *et al.*, 1995 ▸). Information regarding non-covalent bonding present in the crystal is found in Table 1 ▸.

## 4. Database survey

A search in the Cambridge Structural Database (CSD, Version 5.43, update November 2021; Groom *et al.*, 2016 ▸) for 2,4,6-tri­ethyl­benzene derivatives bearing the (4,6-di­methyl­pyridin-2-yl)amino­methyl unit gave eight hits. In the crystal structures of the monohydrate and the methanol solvate of {1-[(3,5-bis­{[(4,6-di­methyl­pyridin-2-yl)amino]­meth­yl}-2,4,6-tri­ethyl­benz­yl)amino]­cyclo­pent­yl}methanol (CADTAG, CADTEK; Stapf *et al.*, 2020*a* ▸), the host mol­ecules reveal similar geometries with an alternating arrangement of the substituents above and below the plane of the central benzene ring. The crystals of these solvates are composed of inversion-symmetric dimers of 1:1 host–guest complexes held together by O—H⋯N and N—H⋯O hydrogen bonds.

In the case of the ethanol solvate of 1,3,5-tris­[(4,6-di­meth­yl­pyridin-2-yl)amino­meth­yl]-2,4,6-tri­ethyl­benzene (RAJ­ZAE; Mazik *et al.*, 2004 ▸), dimers of host–guest units stabilized by O—H⋯N_pyr_ and N—H⋯O bonds represent the basic supra­molecular aggregates. The latter compound is also capable of forming crystalline complexes with methyl β-d-gluco­pyran­oside (LAJZOP; Köhler *et al.*, 2020 ▸). This crystal structure (aceto­nitrile tetra­solvate monohydrate) contains two structurally different 2:1 receptor-carbohydrate complexes in which the sugar substrate is located in a cavity formed by the functionalized side arms of a pair of receptor mol­ecules.

In the crystal structure of 1-{[*N*-(1,10-phenanthrolin-2-ylcarbon­yl)amino]­meth­yl}-3,5-bis­{[(4,6-di­methyl­pyridin-2-yl)amino]­meth­yl}-2,4,6-tri­ethyl­benzene (ROKJEH; Mazik *et al.*, 2008 ▸), three water mol­ecules are accommodated in the binding pocket created by the heterocyclic units (one phenanthrolinyl and two pyridinyl groups) of the host mol­ecule. This host–water aggregate is stabilized by O—H⋯O, N—H⋯O and O—H⋯N hydrogen bonds. In a similar way, two water mol­ecules and one ethanol mol­ecule are accommodated in the binding pocket of 1,3-bis­{[*N*-(1,10-phenanthrolin-2-ylcarbon­yl)amino]­meth­yl}-5-{[(4,6-di­methyl­pyridin-2-yl)amino]­meth­yl}-2,4,6-tri­ethyl­benzene (TUGVEX; Mazik *et al.*, 2009 ▸), containing one pyridinyl and two phenanthrolinyl groups.

## 5. Synthesis and crystallization

A mixture of di­ethano­lamine (0.18 mL, 0.20 g, 1.88 mmol), THF (10 mL) and potassium carbonate (86 mg, 0.62 mmol) was stirred at room temperature for 30 minutes. After that, a solution of 1,3-bis­(bromo­meth­yl)-5-{[(4,6-di­methyl­pyridin-2-yl)amino]­meth­yl}-2,4,6-tri­ethyl­benzene (150 mg, 0.31 mmol) in 10 mL of THF/CH_3_CN (1:1) was added dropwise and the resulting mixture was stirred at room temperature and under light exclusion (the progress of the reaction was monitored by TLC). After filtration, the solvents were removed under reduced pressure and the residual yellow oil was treated with a THF/water mixture. Then the THF was evaporated and the resulting oil was separated from the water. The oil was again dissolved in THF and dried over MgSO_4_. By addition of *n*-hexane, the product was precipitated as a white solid (58% yield, 95 mg, 0.18 mmol). *Analysis data*: m.p. = 408 K; ^1^H NMR (500 MHz, CDCl_3_) *δ* 1.14 (*t*, *J* = 7.5 Hz, 3H, C*H*_3_), 1.19 (*t*, *J* = 7.5 Hz, 6H, C*H*_3_), 2.29 (*s*, 3H, C*H*_3_), 2.35 (*s*, 3H, C*H*_3_), 2.63 (*t*, *J* = 5.0 Hz, 8H, C*H*_2_), 2.80 (*q*, *J* = 7.5 Hz, 4H, C*H*_2_), 3.26 (*q*, *J* = 7.5 Hz, 2H, C*H*_2_), 3.50 (*t*, *J* = 5.0 Hz, 8H, C*H*_2_), 3.77 (*s*, 4H, C*H*_2_), 4.22 (*d*, *J* = 4.0 Hz, 2H, C*H*_2_), 4.60 (*br*, 1H, N*H*), 6.17 (*s*, 1H, Ar*H*), 6.38 (*s*, 1H, Ar*H*); ^13^C NMR (126 MHz, CDCl_3_) *δ* 15.7, 16.5, 21.2, 21.3, 22.9, 23.8, 40.8, 52.3, 55.2, 59.7, 102.7, 114.1, 131.6, 132.6, 143.5, 145.8, 149.7, 156.2, 158.0; MS (ESI): *m*/*z* calculated for C_30_H_51_N_4_O_4_: 531.4 [*M* + H]^+^, found 531.4; *R_f_* = 0.50 (Al_2_O_3_, CHCl_3_/CH_3_OH 7:1). Crystals of the title compound suitable for X-ray analysis were obtained as colourless blocks by diffusion of *n*-hexane into a solution of the compound in THF.

## 6. Refinement

Crystal data, data collection and structure refinement details are summarized in Table 2 ▸. Carbon-bound hydrogen atoms and protons of the minor (12%) positions of the disordered OH groups (H1*A*, H2*A*) were positioned geometrically and allowed to ride on their respective parent atoms, with C—H = 0.95 Å (aromatic) and 0.99 Å (methyl­ene) and *U*_iso_(H) = 1.2 *U*_eq_(C), and O—H = 0.84 Å (OH) and C—H = 0.98 Å (meth­yl) and *U*_iso_(H) = 1.5 *U*_eq_(C,O), respectively. The protons of the N—H and O—H (undisordered or the main positions) were located from the residual electron density map and refined with *U*_iso_(H) bound to the parent atom (1.2, for NH and 1.5 for OH) with distance restraints for the OH bonds (SADI). The refinement of the disordered N(CH_2_CH_2_OH)_2_ group was performed using geometry (SAME) and *U*_ij_ (SIMU, RIGU) restrains implemented in *SHELXL* (Sheldrick, 2015 ▸). The refined proportion of the two positions is 88:12%. The maximum residual peak of 0.55 e Å^−3^ is located inside a 27.5 Å^3^ void and can be refined as a partially occupied water mol­ecule (∼6%); however, due to the low occupancy, it was not included in the final refinement.

## Supplementary Material

Crystal structure: contains datablock(s) I. DOI: 10.1107/S2056989022007411/jq2019sup1.cif

Structure factors: contains datablock(s) I. DOI: 10.1107/S2056989022007411/jq2019Isup2.hkl

Supporting information file. DOI: 10.1107/S2056989022007411/jq2019Isup3.cml

CCDC reference: 2191249

Additional supporting information:  crystallographic information; 3D view; checkCIF report

## Figures and Tables

**Table 1:** Hydrogen-bond geometry (Å, °)

*D*—H⋯*A*	*D*—H	H⋯*A*	*D*⋯*A*	*D*—H⋯*A*
O2*A*—H2*A*⋯O4^i^	0.84	1.99	2.763 (18)	152
O1*A*—H1*A*⋯N2^ii^	0.84	1.99	2.832 (12)	178
C18*A*—H18*D*⋯O2*A*	0.99	2.46	3.08 (2)	120
O2—H2⋯O3^i^	0.87 (2)	1.99 (2)	2.828 (2)	162 (3)
O1—H1⋯N2^ii^	0.87 (2)	1.89 (2)	2.7449 (19)	167 (3)
N1—H1*N*⋯O1^ii^	0.89 (2)	2.19 (2)	3.014 (2)	152.0 (17)
C22—H22*A*⋯N4	0.99	2.40	3.152 (2)	132
C18—H18*A*⋯O2	0.99	2.39	3.106 (3)	128
C15—H15*A*⋯N3	0.99	2.54	3.282 (3)	131
C13—H13*A*⋯O2*A* ^iii^	0.98	2.33	3.220 (14)	151
C10—H10⋯O4^iv^	0.95	2.45	3.365 (2)	161
O4—H4⋯O3	0.86 (2)	2.12 (2)	2.9200 (18)	155 (3)
O3—H3⋯O1*A*	0.87 (2)	1.93 (2)	2.727 (13)	152 (3)
O3—H3⋯O1	0.87 (2)	1.86 (2)	2.7156 (19)	172 (3)

Symmetry codes: (i) 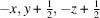 ; (ii) 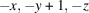 ; (iii) 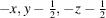 ; (iv) 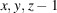 .

**Table 2:** Experimental details

Crystal data	
Chemical formula	C_30_H_50_N_4_O_4_
*M*_r_	530.74
Crystal system, space group	Monoclinic, *P*2_1_/*c*
Temperature (K)	100
*a*, *b*, *c* (Å)	14.2508 (3), 15.3046 (4), 15.2593 (3)
β (°)	113.3107 (13)
*V* (Å^3^)	3056.43 (12)
*Z*	4
Radiation type	Mo *K*α
μ (mm^−1^)	0.08
Crystal size (mm)	0.20 × 0.13 × 0.12

Data collection	
Diffractometer	Bruker Kappa APEXII with CCD area detector
No. of measured, independent and observed [*I* > 2σ(*I*)] reflections	22458, 6881, 4968
*R*_int_	0.031
(sin θ/λ)_max_ (Å^−1^)	0.647

Refinement	
*R*[*F* ^2^ > 2σ(*F* ^2^)], *wR*(*F* ^2^), *S*	0.049, 0.144, 1.03
No. of reflections	6881
No. of parameters	431
No. of restraints	290
H-atom treatment	H atoms treated by a mixture of independent and constrained refinement
Δρ_max_, Δρ_min_ (e Å^−3^)	0.55, −0.26

Computer programs: *APEX2* and *SAINT* (Bruker, 2008 ▸), *SHELXS97* and *SHELXTL* (Sheldrick, 2008 ▸), *SHELXL2018/3* (Sheldrick, 2015 ▸) and *ORTEP-3 for Windows* (Farrugia, 2012 ▸).

## supplementary crystallographic information

### Crystal data

**Table:**

C_30_H_50_N_4_O_4_	*F*(000) = 1160
*M**_r_* = 530.74	*D*_x_ = 1.153 Mg m^−^^3^
Monoclinic, *P*2_1_/*c*	Mo *K*α radiation, λ = 0.71073 Å
*a* = 14.2508 (3) Å	Cell parameters from 5468 reflections
*b* = 15.3046 (4) Å	θ = 2.7–28.2°
*c* = 15.2593 (3) Å	µ = 0.08 mm^−^^1^
β = 113.3107 (13)°	*T* = 100 K
*V* = 3056.43 (12) Å^3^	Rod, colourless
*Z* = 4	0.20 × 0.13 × 0.12 mm

### Data collection

**Table:**

Bruker Kappa APEXII with CCD area detector diffractometer	*R*_int_ = 0.031
Radiation source: fine-focus sealed X-Ray tube	θ_max_ = 27.4°, θ_min_ = 3.1°
phi and ω scans	*h* = −18→17
22458 measured reflections	*k* = −19→12
6881 independent reflections	*l* = −19→19
4968 reflections with *I* > 2σ(*I*)	

### Refinement

**Table:**

Refinement on *F*^2^	Primary atom site location: structure-invariant direct methods
Least-squares matrix: full	Secondary atom site location: difference Fourier map
*R*[*F*^2^ > 2σ(*F*^2^)] = 0.049	Hydrogen site location: mixed
*wR*(*F*^2^) = 0.144	H atoms treated by a mixture of independent and constrained refinement
*S* = 1.03	*w* = 1/[σ^2^(*F*_o_^2^) + (0.0773*P*)^2^ + 0.7833*P*] where *P* = (*F*_o_^2^ + 2*F*_c_^2^)/3
6881 reflections	(Δ/σ)_max_ = 0.001
431 parameters	Δρ_max_ = 0.55 e Å^−^^3^
290 restraints	Δρ_min_ = −0.26 e Å^−^^3^

### Special details

**Table:**

Geometry. All esds (except the esd in the dihedral angle between two l.s. planes) are estimated using the full covariance matrix. The cell esds are taken into account individually in the estimation of esds in distances, angles and torsion angles; correlations between esds in cell parameters are only used when they are defined by crystal symmetry. An approximate (isotropic) treatment of cell esds is used for estimating esds involving l.s. planes.

### Fractional atomic coordinates and isotropic or equivalent isotropic displacement parameters (Å^2^)

**Table:**

	*x*	*y*	*z*	*U*_iso_*/*U*_eq_	Occ. (<1)
O3	0.06213 (10)	0.37633 (9)	0.20622 (9)	0.0317 (3)	
H3	0.0307 (19)	0.4220 (14)	0.1746 (17)	0.066 (8)*	
O4	0.14319 (11)	0.36916 (9)	0.41431 (9)	0.0344 (3)	
H4	0.133 (2)	0.3843 (18)	0.3569 (14)	0.075 (9)*	
N1	0.27113 (11)	0.44288 (9)	−0.07113 (9)	0.0208 (3)	
H1N	0.2143 (16)	0.4558 (13)	−0.0622 (13)	0.031 (5)*	
N2	0.15327 (11)	0.43433 (8)	−0.22623 (9)	0.0228 (3)	
N4	0.27759 (10)	0.34248 (8)	0.32090 (9)	0.0205 (3)	
C1	0.35480 (12)	0.49260 (10)	0.09155 (10)	0.0187 (3)	
C2	0.31562 (12)	0.57360 (10)	0.10358 (11)	0.0194 (3)	
C3	0.30179 (12)	0.59065 (9)	0.18829 (11)	0.0181 (3)	
C4	0.32351 (11)	0.52609 (10)	0.25834 (10)	0.0173 (3)	
C5	0.35813 (12)	0.44317 (9)	0.24358 (10)	0.0174 (3)	
C6	0.37449 (11)	0.42637 (10)	0.16047 (10)	0.0177 (3)	
C7	0.36773 (12)	0.47485 (11)	−0.00056 (11)	0.0221 (3)	
H7A	0.387939	0.529168	−0.023767	0.027*	
H7B	0.421962	0.430698	0.010295	0.027*	
C8	0.25215 (12)	0.43984 (9)	−0.16689 (11)	0.0193 (3)	
C9	0.12953 (14)	0.42942 (11)	−0.32090 (12)	0.0267 (4)	
C10	0.20380 (14)	0.43045 (11)	−0.35800 (12)	0.0275 (4)	
H10	0.185041	0.427754	−0.425011	0.033*	
C11	0.30622 (14)	0.43547 (11)	−0.29619 (12)	0.0273 (4)	
C12	0.33091 (13)	0.43981 (10)	−0.19916 (11)	0.0233 (3)	
H12	0.400235	0.442732	−0.155317	0.028*	
C13	0.01792 (15)	0.42276 (15)	−0.38339 (14)	0.0410 (5)	
H13A	0.009330	0.414806	−0.449864	0.062*	
H13B	−0.016851	0.476413	−0.377828	0.062*	
H13C	−0.011639	0.372699	−0.363317	0.062*	
C14	0.38925 (17)	0.43524 (14)	−0.33408 (15)	0.0413 (5)	
H14A	0.396002	0.376396	−0.356233	0.062*	
H14B	0.454117	0.452731	−0.283249	0.062*	
H14C	0.371554	0.476451	−0.387372	0.062*	
C15	0.28910 (15)	0.64292 (11)	0.02668 (12)	0.0301 (4)	
H15A	0.232164	0.678992	0.028352	0.036*	
H15B	0.265364	0.614087	−0.036485	0.036*	
C16	0.37980 (19)	0.70256 (13)	0.03842 (16)	0.0468 (6)	
H16A	0.435285	0.667612	0.033810	0.070*	
H16B	0.403792	0.731212	0.100914	0.070*	
H16C	0.358268	0.746977	−0.011954	0.070*	
C17	0.25925 (12)	0.67846 (10)	0.20094 (12)	0.0220 (3)	
H17A	0.275654	0.688014	0.269611	0.026*	0.879 (2)
H17B	0.292937	0.725243	0.179147	0.026*	0.879 (2)
H17C	0.295174	0.699548	0.267252	0.026*	0.121 (2)
H17D	0.268160	0.722174	0.157027	0.026*	0.121 (2)
N3	0.14881 (13)	0.68479 (11)	0.14808 (13)	0.0210 (4)	0.879 (2)
C18	0.09000 (15)	0.61856 (13)	0.17436 (13)	0.0257 (4)	0.879 (2)
H18A	0.067231	0.643267	0.222641	0.031*	0.879 (2)
H18B	0.134482	0.567761	0.203334	0.031*	0.879 (2)
C19	−0.0016 (2)	0.5886 (3)	0.0894 (3)	0.0291 (8)	0.879 (2)
H19A	−0.050599	0.637513	0.064946	0.035*	0.879 (2)
H19B	0.019809	0.569992	0.037941	0.035*	0.879 (2)
O1	−0.04980 (13)	0.51746 (9)	0.11566 (11)	0.0291 (4)	0.879 (2)
H1	−0.0746 (19)	0.5386 (16)	0.1552 (16)	0.044*	0.879 (2)
C20	0.11081 (16)	0.77397 (12)	0.14305 (16)	0.0307 (5)	0.879 (2)
H20A	0.035285	0.772108	0.111954	0.037*	0.879 (2)
H20B	0.135047	0.808013	0.100913	0.037*	0.879 (2)
C21	0.1402 (2)	0.82323 (15)	0.2360 (2)	0.0441 (6)	0.879 (2)
H21A	0.215140	0.831891	0.264051	0.053*	0.879 (2)
H21B	0.107765	0.881661	0.222582	0.053*	0.879 (2)
O2	0.11150 (16)	0.78077 (12)	0.30323 (13)	0.0529 (5)	0.879 (2)
H2	0.0511 (16)	0.801 (2)	0.292 (2)	0.088 (12)*	0.879 (2)
N3A	0.1503 (6)	0.6654 (8)	0.1793 (8)	0.028 (3)	0.121 (2)
C18A	0.0841 (8)	0.6365 (8)	0.0834 (7)	0.022 (2)	0.121 (2)
H18C	0.119291	0.590229	0.062551	0.026*	0.121 (2)
H18D	0.071014	0.686176	0.038571	0.026*	0.121 (2)
C19A	−0.0162 (11)	0.6017 (19)	0.0800 (16)	0.025 (4)	0.121 (2)
H19C	−0.061027	0.650470	0.081917	0.030*	0.121 (2)
H19D	−0.051601	0.568134	0.020504	0.030*	0.121 (2)
O1A	0.0064 (10)	0.5463 (9)	0.1615 (10)	0.051 (3)	0.121 (2)
H1A	−0.040111	0.550860	0.181942	0.076*	0.121 (2)
C20A	0.1121 (12)	0.7331 (9)	0.2246 (10)	0.050 (3)	0.121 (2)
H20C	0.152433	0.732286	0.294424	0.060*	0.121 (2)
H20D	0.039976	0.720561	0.213106	0.060*	0.121 (2)
C21A	0.1192 (18)	0.8226 (10)	0.1859 (13)	0.066 (5)	0.121 (2)
H21C	0.106339	0.867512	0.226497	0.079*	0.121 (2)
H21D	0.189389	0.831435	0.189293	0.079*	0.121 (2)
O2A	0.0491 (14)	0.8342 (12)	0.0907 (11)	0.087 (5)	0.121 (2)
H2A	−0.010553	0.825947	0.087201	0.130*	0.121 (2)
C22	0.31398 (13)	0.54528 (10)	0.35242 (11)	0.0229 (3)	
H22A	0.299154	0.490307	0.378670	0.027*	
H22B	0.256090	0.585763	0.340778	0.027*	
C23	0.41152 (14)	0.58586 (11)	0.42515 (11)	0.0261 (4)	
H23A	0.469352	0.546753	0.435284	0.039*	
H23B	0.404018	0.594612	0.485670	0.039*	
H23C	0.423840	0.642269	0.401207	0.039*	
C24	0.37421 (12)	0.37309 (10)	0.31879 (11)	0.0205 (3)	
H24A	0.410543	0.322946	0.305591	0.025*	
H24B	0.417870	0.396841	0.382251	0.025*	
C25	0.21403 (13)	0.29319 (11)	0.23560 (11)	0.0246 (4)	
H25A	0.182595	0.243188	0.255098	0.029*	
H25B	0.258086	0.269381	0.204862	0.029*	
C26	0.13072 (13)	0.34762 (11)	0.16397 (12)	0.0265 (4)	
H26A	0.160910	0.398788	0.145041	0.032*	
H26B	0.093041	0.312534	0.106152	0.032*	
C27	0.29400 (14)	0.29730 (11)	0.41001 (12)	0.0264 (4)	
H27A	0.344684	0.330023	0.463986	0.032*	
H27B	0.322171	0.238398	0.408799	0.032*	
C28	0.19565 (15)	0.28872 (12)	0.42530 (13)	0.0320 (4)	
H28A	0.150549	0.245962	0.379068	0.038*	
H28B	0.211491	0.265817	0.490291	0.038*	
C29	0.41588 (13)	0.33905 (10)	0.14462 (12)	0.0246 (4)	
H29A	0.389151	0.326780	0.075312	0.029*	
H29B	0.391172	0.292370	0.175083	0.029*	
C30	0.53240 (14)	0.33718 (12)	0.18528 (13)	0.0336 (4)	
H30A	0.555528	0.279991	0.172831	0.050*	
H30B	0.559255	0.347470	0.254260	0.050*	
H30C	0.557266	0.382886	0.154862	0.050*	

### Atomic displacement parameters (Å^2^)

**Table:**

	*U*^11^	*U*^22^	*U*^33^	*U*^12^	*U*^13^	*U*^23^
O3	0.0266 (7)	0.0379 (7)	0.0313 (7)	0.0018 (6)	0.0122 (6)	0.0082 (6)
O4	0.0409 (8)	0.0418 (8)	0.0244 (7)	0.0039 (6)	0.0170 (6)	0.0003 (6)
N1	0.0216 (7)	0.0258 (7)	0.0169 (7)	−0.0023 (6)	0.0096 (6)	−0.0033 (5)
N2	0.0267 (8)	0.0207 (7)	0.0218 (7)	0.0009 (5)	0.0103 (6)	0.0008 (5)
N4	0.0258 (7)	0.0192 (6)	0.0153 (6)	−0.0026 (5)	0.0068 (6)	0.0014 (5)
C1	0.0180 (7)	0.0219 (7)	0.0168 (7)	−0.0045 (6)	0.0077 (6)	−0.0025 (6)
C2	0.0188 (8)	0.0188 (7)	0.0194 (8)	−0.0033 (6)	0.0063 (6)	0.0013 (6)
C3	0.0167 (7)	0.0153 (7)	0.0221 (8)	−0.0014 (6)	0.0075 (6)	−0.0011 (6)
C4	0.0169 (7)	0.0172 (7)	0.0188 (7)	−0.0023 (6)	0.0084 (6)	−0.0027 (6)
C5	0.0178 (7)	0.0169 (7)	0.0168 (7)	−0.0005 (6)	0.0062 (6)	0.0000 (6)
C6	0.0167 (7)	0.0182 (7)	0.0183 (7)	−0.0008 (6)	0.0069 (6)	−0.0036 (6)
C7	0.0215 (8)	0.0286 (8)	0.0180 (8)	−0.0041 (7)	0.0096 (7)	−0.0028 (6)
C8	0.0261 (8)	0.0147 (7)	0.0185 (7)	0.0000 (6)	0.0104 (7)	0.0001 (6)
C9	0.0325 (10)	0.0221 (8)	0.0232 (8)	−0.0002 (7)	0.0088 (7)	−0.0002 (6)
C10	0.0395 (10)	0.0268 (9)	0.0174 (8)	−0.0027 (7)	0.0127 (8)	−0.0005 (6)
C11	0.0360 (10)	0.0255 (8)	0.0261 (9)	−0.0049 (7)	0.0184 (8)	−0.0036 (7)
C12	0.0246 (9)	0.0254 (8)	0.0210 (8)	−0.0029 (7)	0.0101 (7)	−0.0038 (6)
C13	0.0350 (11)	0.0513 (12)	0.0294 (10)	0.0004 (9)	0.0047 (9)	0.0011 (9)
C14	0.0483 (13)	0.0514 (12)	0.0367 (11)	−0.0127 (10)	0.0301 (10)	−0.0116 (9)
C15	0.0458 (11)	0.0221 (8)	0.0234 (8)	−0.0009 (8)	0.0145 (8)	0.0052 (7)
C16	0.0773 (17)	0.0288 (10)	0.0509 (13)	−0.0146 (10)	0.0429 (13)	−0.0012 (9)
C17	0.0218 (8)	0.0189 (7)	0.0255 (8)	0.0010 (6)	0.0093 (7)	−0.0014 (6)
N3	0.0214 (9)	0.0169 (8)	0.0247 (9)	0.0047 (6)	0.0092 (7)	0.0066 (7)
C18	0.0249 (10)	0.0268 (10)	0.0260 (10)	0.0008 (8)	0.0109 (8)	0.0009 (8)
C19	0.0286 (13)	0.0305 (17)	0.0260 (13)	0.0004 (12)	0.0085 (10)	0.0001 (10)
O1	0.0237 (8)	0.0311 (8)	0.0342 (8)	0.0008 (6)	0.0130 (7)	−0.0026 (6)
C20	0.0276 (11)	0.0183 (9)	0.0487 (13)	0.0081 (8)	0.0178 (10)	0.0071 (9)
C21	0.0336 (14)	0.0276 (11)	0.0711 (18)	0.0065 (10)	0.0207 (14)	−0.0135 (12)
O2	0.0628 (13)	0.0487 (10)	0.0471 (10)	0.0246 (9)	0.0218 (10)	−0.0080 (8)
N3A	0.025 (5)	0.018 (5)	0.035 (5)	−0.001 (4)	0.006 (4)	−0.002 (4)
C18A	0.013 (4)	0.020 (5)	0.027 (5)	0.003 (4)	0.002 (4)	−0.004 (4)
C19A	0.019 (6)	0.021 (7)	0.033 (7)	−0.005 (5)	0.008 (5)	−0.014 (5)
O1A	0.026 (6)	0.069 (8)	0.053 (7)	0.011 (6)	0.011 (5)	0.021 (6)
C20A	0.046 (6)	0.042 (5)	0.054 (6)	0.008 (5)	0.010 (5)	−0.012 (5)
C21A	0.060 (8)	0.047 (7)	0.077 (8)	0.007 (6)	0.013 (7)	0.004 (6)
O2A	0.077 (10)	0.096 (11)	0.080 (8)	0.027 (8)	0.023 (7)	0.017 (7)
C22	0.0326 (9)	0.0182 (7)	0.0236 (8)	−0.0005 (7)	0.0174 (7)	−0.0018 (6)
C23	0.0374 (10)	0.0228 (8)	0.0195 (8)	0.0007 (7)	0.0126 (7)	−0.0035 (6)
C24	0.0243 (8)	0.0183 (7)	0.0173 (7)	0.0015 (6)	0.0066 (7)	0.0012 (6)
C25	0.0284 (9)	0.0214 (8)	0.0227 (8)	−0.0037 (7)	0.0088 (7)	−0.0026 (6)
C26	0.0259 (9)	0.0329 (9)	0.0197 (8)	−0.0065 (7)	0.0077 (7)	−0.0001 (7)
C27	0.0341 (10)	0.0245 (8)	0.0195 (8)	−0.0002 (7)	0.0094 (7)	0.0060 (6)
C28	0.0398 (11)	0.0338 (10)	0.0249 (9)	−0.0032 (8)	0.0153 (8)	0.0042 (7)
C29	0.0306 (9)	0.0212 (8)	0.0230 (8)	0.0032 (7)	0.0118 (7)	−0.0040 (6)
C30	0.0323 (10)	0.0331 (9)	0.0348 (10)	0.0130 (8)	0.0128 (8)	−0.0021 (8)

### Geometric parameters (Å, º)

**Table:**

O3—C26	1.436 (2)	C18—C19	1.502 (4)
O3—H3	0.867 (16)	C18—H18A	0.9900
O4—C28	1.415 (2)	C18—H18B	0.9900
O4—H4	0.863 (16)	C19—O1	1.427 (3)
N1—C8	1.3775 (19)	C19—H19A	0.9900
N1—C7	1.456 (2)	C19—H19B	0.9900
N1—H1N	0.89 (2)	O1—H1	0.873 (16)
N2—C8	1.343 (2)	C20—C21	1.512 (3)
N2—C9	1.349 (2)	C20—H20A	0.9900
N4—C27	1.4595 (19)	C20—H20B	0.9900
N4—C25	1.466 (2)	C21—O2	1.404 (3)
N4—C24	1.467 (2)	C21—H21A	0.9900
C1—C2	1.401 (2)	C21—H21B	0.9900
C1—C6	1.407 (2)	O2—H2	0.865 (17)
C1—C7	1.513 (2)	N3A—C18A	1.461 (9)
C2—C3	1.407 (2)	N3A—C20A	1.466 (9)
C2—C15	1.515 (2)	C18A—C19A	1.507 (10)
C3—C4	1.397 (2)	C18A—H18C	0.9900
C3—C17	1.518 (2)	C18A—H18D	0.9900
C4—C5	1.412 (2)	C19A—O1A	1.433 (10)
C4—C22	1.523 (2)	C19A—H19C	0.9900
C5—C6	1.4010 (19)	C19A—H19D	0.9900
C5—C24	1.521 (2)	O1A—H1A	0.8400
C6—C29	1.518 (2)	C20A—C21A	1.511 (10)
C7—H7A	0.9900	C20A—H20C	0.9900
C7—H7B	0.9900	C20A—H20D	0.9900
C8—C12	1.393 (2)	C21A—O2A	1.411 (10)
C9—C10	1.384 (2)	C21A—H21C	0.9900
C9—C13	1.500 (3)	C21A—H21D	0.9900
C10—C11	1.392 (3)	O2A—H2A	0.8400
C10—H10	0.9500	C22—C23	1.526 (2)
C11—C12	1.381 (2)	C22—H22A	0.9900
C11—C14	1.509 (2)	C22—H22B	0.9900
C12—H12	0.9500	C23—H23A	0.9800
C13—H13A	0.9800	C23—H23B	0.9800
C13—H13B	0.9800	C23—H23C	0.9800
C13—H13C	0.9800	C24—H24A	0.9900
C14—H14A	0.9800	C24—H24B	0.9900
C14—H14B	0.9800	C25—C26	1.507 (2)
C14—H14C	0.9800	C25—H25A	0.9900
C15—C16	1.534 (3)	C25—H25B	0.9900
C15—H15A	0.9900	C26—H26A	0.9900
C15—H15B	0.9900	C26—H26B	0.9900
C16—H16A	0.9800	C27—C28	1.515 (2)
C16—H16B	0.9800	C27—H27A	0.9900
C16—H16C	0.9800	C27—H27B	0.9900
C17—N3	1.460 (2)	C28—H28A	0.9900
C17—N3A	1.468 (8)	C28—H28B	0.9900
C17—H17A	0.9900	C29—C30	1.526 (2)
C17—H17B	0.9900	C29—H29A	0.9900
C17—H17C	0.9900	C29—H29B	0.9900
C17—H17D	0.9900	C30—H30A	0.9800
N3—C20	1.459 (2)	C30—H30B	0.9800
N3—C18	1.469 (2)	C30—H30C	0.9800
			
C26—O3—H3	106.9 (18)	H19A—C19—H19B	108.2
C28—O4—H4	102.7 (19)	C19—O1—H1	106.4 (17)
C8—N1—C7	121.81 (13)	N3—C20—C21	117.25 (18)
C8—N1—H1N	111.1 (12)	N3—C20—H20A	108.0
C7—N1—H1N	117.4 (12)	C21—C20—H20A	108.0
C8—N2—C9	118.51 (14)	N3—C20—H20B	108.0
C27—N4—C25	113.46 (12)	C21—C20—H20B	108.0
C27—N4—C24	111.47 (13)	H20A—C20—H20B	107.2
C25—N4—C24	113.60 (12)	O2—C21—C20	113.70 (19)
C2—C1—C6	120.77 (13)	O2—C21—H21A	108.8
C2—C1—C7	118.81 (13)	C20—C21—H21A	108.8
C6—C1—C7	120.24 (13)	O2—C21—H21B	108.8
C1—C2—C3	119.50 (13)	C20—C21—H21B	108.8
C1—C2—C15	120.56 (13)	H21A—C21—H21B	107.7
C3—C2—C15	119.94 (14)	C21—O2—H2	104 (2)
C4—C3—C2	120.16 (13)	C18A—N3A—C20A	118.3 (9)
C4—C3—C17	120.50 (13)	C18A—N3A—C17	118.2 (8)
C2—C3—C17	119.29 (13)	C20A—N3A—C17	110.8 (8)
C3—C4—C5	119.95 (13)	N3A—C18A—C19A	111.7 (11)
C3—C4—C22	120.61 (13)	N3A—C18A—H18C	109.3
C5—C4—C22	119.40 (13)	C19A—C18A—H18C	109.3
C6—C5—C4	120.22 (13)	N3A—C18A—H18D	109.3
C6—C5—C24	121.63 (13)	C19A—C18A—H18D	109.3
C4—C5—C24	118.13 (12)	H18C—C18A—H18D	107.9
C5—C6—C1	119.28 (13)	O1A—C19A—C18A	107.1 (10)
C5—C6—C29	121.33 (13)	O1A—C19A—H19C	110.3
C1—C6—C29	119.37 (12)	C18A—C19A—H19C	110.3
N1—C7—C1	108.68 (12)	O1A—C19A—H19D	110.3
N1—C7—H7A	110.0	C18A—C19A—H19D	110.3
C1—C7—H7A	110.0	H19C—C19A—H19D	108.5
N1—C7—H7B	110.0	C19A—O1A—H1A	109.5
C1—C7—H7B	110.0	N3A—C20A—C21A	111.2 (12)
H7A—C7—H7B	108.3	N3A—C20A—H20C	109.4
N2—C8—N1	115.51 (13)	C21A—C20A—H20C	109.4
N2—C8—C12	122.62 (14)	N3A—C20A—H20D	109.4
N1—C8—C12	121.84 (15)	C21A—C20A—H20D	109.4
N2—C9—C10	121.94 (16)	H20C—C20A—H20D	108.0
N2—C9—C13	115.99 (15)	O2A—C21A—C20A	112.8 (12)
C10—C9—C13	122.07 (16)	O2A—C21A—H21C	109.0
C9—C10—C11	119.35 (15)	C20A—C21A—H21C	109.0
C9—C10—H10	120.3	O2A—C21A—H21D	109.0
C11—C10—H10	120.3	C20A—C21A—H21D	109.0
C12—C11—C10	118.89 (15)	H21C—C21A—H21D	107.8
C12—C11—C14	120.34 (17)	C21A—O2A—H2A	109.5
C10—C11—C14	120.76 (15)	C4—C22—C23	111.64 (13)
C11—C12—C8	118.68 (16)	C4—C22—H22A	109.3
C11—C12—H12	120.7	C23—C22—H22A	109.3
C8—C12—H12	120.7	C4—C22—H22B	109.3
C9—C13—H13A	109.5	C23—C22—H22B	109.3
C9—C13—H13B	109.5	H22A—C22—H22B	108.0
H13A—C13—H13B	109.5	C22—C23—H23A	109.5
C9—C13—H13C	109.5	C22—C23—H23B	109.5
H13A—C13—H13C	109.5	H23A—C23—H23B	109.5
H13B—C13—H13C	109.5	C22—C23—H23C	109.5
C11—C14—H14A	109.5	H23A—C23—H23C	109.5
C11—C14—H14B	109.5	H23B—C23—H23C	109.5
H14A—C14—H14B	109.5	N4—C24—C5	112.30 (13)
C11—C14—H14C	109.5	N4—C24—H24A	109.1
H14A—C14—H14C	109.5	C5—C24—H24A	109.1
H14B—C14—H14C	109.5	N4—C24—H24B	109.1
C2—C15—C16	112.70 (16)	C5—C24—H24B	109.1
C2—C15—H15A	109.1	H24A—C24—H24B	107.9
C16—C15—H15A	109.1	N4—C25—C26	113.08 (14)
C2—C15—H15B	109.1	N4—C25—H25A	109.0
C16—C15—H15B	109.1	C26—C25—H25A	109.0
H15A—C15—H15B	107.8	N4—C25—H25B	109.0
C15—C16—H16A	109.5	C26—C25—H25B	109.0
C15—C16—H16B	109.5	H25A—C25—H25B	107.8
H16A—C16—H16B	109.5	O3—C26—C25	108.92 (13)
C15—C16—H16C	109.5	O3—C26—H26A	109.9
H16A—C16—H16C	109.5	C25—C26—H26A	109.9
H16B—C16—H16C	109.5	O3—C26—H26B	109.9
N3—C17—C3	112.70 (14)	C25—C26—H26B	109.9
N3A—C17—C3	106.9 (5)	H26A—C26—H26B	108.3
N3—C17—H17A	109.1	N4—C27—C28	111.58 (14)
C3—C17—H17A	109.1	N4—C27—H27A	109.3
N3—C17—H17B	109.1	C28—C27—H27A	109.3
C3—C17—H17B	109.1	N4—C27—H27B	109.3
H17A—C17—H17B	107.8	C28—C27—H27B	109.3
N3A—C17—H17C	110.3	H27A—C27—H27B	108.0
C3—C17—H17C	110.3	O4—C28—C27	112.57 (14)
N3A—C17—H17D	110.3	O4—C28—H28A	109.1
C3—C17—H17D	110.3	C27—C28—H28A	109.1
H17C—C17—H17D	108.6	O4—C28—H28B	109.1
C20—N3—C17	112.80 (16)	C27—C28—H28B	109.1
C20—N3—C18	114.82 (16)	H28A—C28—H28B	107.8
C17—N3—C18	114.22 (15)	C6—C29—C30	112.38 (14)
N3—C18—C19	111.71 (17)	C6—C29—H29A	109.1
N3—C18—H18A	109.3	C30—C29—H29A	109.1
C19—C18—H18A	109.3	C6—C29—H29B	109.1
N3—C18—H18B	109.3	C30—C29—H29B	109.1
C19—C18—H18B	109.3	H29A—C29—H29B	107.9
H18A—C18—H18B	107.9	C29—C30—H30A	109.5
O1—C19—C18	109.9 (2)	C29—C30—H30B	109.5
O1—C19—H19A	109.7	H30A—C30—H30B	109.5
C18—C19—H19A	109.7	C29—C30—H30C	109.5
O1—C19—H19B	109.7	H30A—C30—H30C	109.5
C18—C19—H19B	109.7	H30B—C30—H30C	109.5
			
C6—C1—C2—C3	3.9 (2)	N2—C8—C12—C11	1.1 (2)
C7—C1—C2—C3	179.11 (14)	N1—C8—C12—C11	179.13 (15)
C6—C1—C2—C15	−176.63 (15)	C1—C2—C15—C16	−88.89 (19)
C7—C1—C2—C15	−1.5 (2)	C3—C2—C15—C16	90.55 (19)
C1—C2—C3—C4	−2.3 (2)	C4—C3—C17—N3	−99.98 (17)
C15—C2—C3—C4	178.26 (15)	C2—C3—C17—N3	77.67 (18)
C1—C2—C3—C17	−179.95 (14)	C4—C3—C17—N3A	−77.6 (6)
C15—C2—C3—C17	0.6 (2)	C2—C3—C17—N3A	100.1 (6)
C2—C3—C4—C5	−0.8 (2)	C3—C17—N3—C20	−167.61 (15)
C17—C3—C4—C5	176.79 (14)	C3—C17—N3—C18	58.88 (19)
C2—C3—C4—C22	176.88 (14)	C20—N3—C18—C19	83.7 (3)
C17—C3—C4—C22	−5.5 (2)	C17—N3—C18—C19	−143.8 (2)
C3—C4—C5—C6	2.4 (2)	N3—C18—C19—O1	173.3 (2)
C22—C4—C5—C6	−175.35 (14)	C17—N3—C20—C21	−52.4 (2)
C3—C4—C5—C24	−176.09 (14)	C18—N3—C20—C21	80.8 (2)
C22—C4—C5—C24	6.2 (2)	N3—C20—C21—O2	−56.8 (3)
C4—C5—C6—C1	−0.8 (2)	C3—C17—N3A—C18A	−61.6 (11)
C24—C5—C6—C1	177.64 (14)	C3—C17—N3A—C20A	157.2 (8)
C4—C5—C6—C29	177.43 (14)	C20A—N3A—C18A—C19A	−58.0 (19)
C24—C5—C6—C29	−4.1 (2)	C17—N3A—C18A—C19A	163.7 (14)
C2—C1—C6—C5	−2.4 (2)	N3A—C18A—C19A—O1A	−45 (3)
C7—C1—C6—C5	−177.49 (14)	C18A—N3A—C20A—C21A	−77.9 (15)
C2—C1—C6—C29	179.35 (14)	C17—N3A—C20A—C21A	63.2 (15)
C7—C1—C6—C29	4.2 (2)	N3A—C20A—C21A—O2A	70 (2)
C8—N1—C7—C1	165.06 (14)	C3—C4—C22—C23	−85.93 (18)
C2—C1—C7—N1	−85.21 (17)	C5—C4—C22—C23	91.81 (17)
C6—C1—C7—N1	90.00 (17)	C27—N4—C24—C5	−162.08 (12)
C9—N2—C8—N1	−178.75 (14)	C25—N4—C24—C5	68.22 (16)
C9—N2—C8—C12	−0.6 (2)	C6—C5—C24—N4	−108.97 (16)
C7—N1—C8—N2	−161.06 (14)	C4—C5—C24—N4	69.50 (18)
C7—N1—C8—C12	20.8 (2)	C27—N4—C25—C26	132.61 (15)
C8—N2—C9—C10	−0.4 (2)	C24—N4—C25—C26	−98.70 (15)
C8—N2—C9—C13	179.58 (15)	N4—C25—C26—O3	−63.25 (17)
N2—C9—C10—C11	0.9 (2)	C25—N4—C27—C28	−66.62 (18)
C13—C9—C10—C11	−179.07 (17)	C24—N4—C27—C28	163.61 (13)
C9—C10—C11—C12	−0.4 (2)	N4—C27—C28—O4	−50.53 (19)
C9—C10—C11—C14	179.00 (16)	C5—C6—C29—C30	−88.66 (18)
C10—C11—C12—C8	−0.6 (2)	C1—C6—C29—C30	89.57 (18)
C14—C11—C12—C8	−179.97 (15)		

### Hydrogen-bond geometry (Å, º)

**Table:**

*D*—H···*A*	*D*—H	H···*A*	*D*···*A*	*D*—H···*A*
O2*A*—H2*A*···O4^i^	0.84	1.99	2.763 (18)	152
O1*A*—H1*A*···N2^ii^	0.84	1.99	2.832 (12)	178
C18*A*—H18*D*···O2*A*	0.99	2.46	3.08 (2)	120
O2—H2···O3^i^	0.87 (2)	1.99 (2)	2.828 (2)	162 (3)
O1—H1···N2^ii^	0.87 (2)	1.89 (2)	2.7449 (19)	167 (3)
N1—H1*N*···O1^ii^	0.89 (2)	2.19 (2)	3.014 (2)	152.0 (17)
C22—H22*A*···N4	0.99	2.40	3.152 (2)	132
C18—H18*A*···O2	0.99	2.39	3.106 (3)	128
C15—H15*A*···N3	0.99	2.54	3.282 (3)	131
C13—H13*A*···O2*A*^iii^	0.98	2.33	3.220 (14)	151
C10—H10···O4^iv^	0.95	2.45	3.365 (2)	161
O4—H4···O3	0.86 (2)	2.12 (2)	2.9200 (18)	155 (3)
O3—H3···O1*A*	0.87 (2)	1.93 (2)	2.727 (13)	152 (3)
O3—H3···O1	0.87 (2)	1.86 (2)	2.7156 (19)	172 (3)

Symmetry codes: (i) −*x*, *y*+1/2, −*z*+1/2; (ii) −*x*, −*y*+1, −*z*; (iii) −*x*, *y*−1/2, −*z*−1/2; (iv) *x*, *y*, *z*−1.

## References

[bb1] Amrhein, F., Lippe, J. & Mazik, M. (2016). *Org. Biomol. Chem.* **14**, 10648–10659. 10.1039/c6ob01682k 27782281

[bb2] Amrhein, F. & Mazik, M. (2021). *Eur. J. Org. Chem.* pp. 6282–6303.

[bb3] Bernstein, J., Davis, R. E., Shimoni, L. & Chang, N.-L. (1995). *Angew. Chem. Int. Ed. Engl.* **34**, 1555–1573.

[bb4] Bruker (2008). *APEX2* and *SAINT*. Bruker AXS Inc., Madison, Wisconsin, USA.

[bb5] Desiraju, G. R. & Steiner, T. (1999). *The Weak Hydrogen Bond In Structural Chemistry and Biology, IUCr Monographs on Crystallography*, Vol. 9. New York: Oxford University Press.

[bb6] Etter, M. C. (1990). *Acc. Chem. Res.* **23**, 120–126.

[bb7] Farrugia, L. J. (2012). *J. Appl. Cryst.* **45**, 849–854.

[bb8] Geffert, C., Kuschel, M. & Mazik, M. (2013). *J. Org. Chem.* **78**, 292–300. 10.1021/jo301966z 23270379

[bb9] Groom, C. R., Bruno, I. J., Lightfoot, M. P. & Ward, S. C. (2016). *Acta Cryst.* B**72**, 171–179. 10.1107/S2052520616003954 PMC4822653 27048719

[bb10] Hennrich, G. & Anslyn, E. V. (2002). *Chem. Eur. J.* **8**, 2218–2224. 10.1002/1521-3765(20020517)8:10<2218::AID-CHEM2218>3.0.CO;2-H 12012405

[bb11] Kaiser, S., Geffert, C. & Mazik, M. (2019). *Eur. J. Org. Chem.* pp. 7555–7562.

[bb12] Koch, N., Seichter, W. & Mazik, M. (2016). *Synthesis*, **48**, 2757–2767.

[bb13] Köhler, L., Hübler, C., Seichter, W. & Mazik, M. (2021). *RSC Adv.* **11**, 22221–22229. 10.1039/d1ra03390e PMC9034237 35480817

[bb14] Köhler, L., Seichter, W. & Mazik, M. (2020). *Eur. J. Org. Chem.* pp. 7023–7034.

[bb15] Lippe, J. & Mazik, M. (2015). *J. Org. Chem.* **80**, 1427–1439. 10.1021/jo502335u 25531805

[bb16] Lippe, J., Seichter, W. & Mazik, M. (2015). *Org. Biomol. Chem.* **13**, 11622–11632. 10.1039/c5ob01757b 26467387

[bb17] Mazik, M. (2009). *Chem. Soc. Rev.* **38**, 935–956. 10.1039/b710910p 19421573

[bb18] Mazik, M. (2012). *RSC Adv.* **2**, 2630–2642.

[bb19] Mazik, M. & Hartmann, A. (2008). *J. Org. Chem.* **73**, 7444–7450. 10.1021/jo8005842 18576685

[bb20] Mazik, M., Hartmann, A. & Jones, P. G. (2009). *Chem. Eur. J.* **15**, 9147–9159. 10.1002/chem.200900664 19650090

[bb21] Mazik, M., Radunz, W. & Boese, R. (2004). *J. Org. Chem.* **69**, 7448–7462. 10.1021/jo048979k 15497969

[bb31] Nishio, M., Umezawa, Y., Hirota, M. & Takeuchi, Y. (1995). *Tetrahedron*, **51**, 8665–8701.

[bb22] Quiocho, F. A. (1989). *Pure Appl. Chem.* **61**, 1293–1306.

[bb23] Rosien, J.-R., Seichter, W. & Mazik, M. (2013). *Org. Biomol. Chem.* **11**, 6569–6579. 10.1039/c3ob41540f 23982815

[bb24] Schulze, M. M., Koch, N., Seichter, W. & Mazik, M. (2018). *Eur. J. Org. Chem.* pp. 4317–4330.

[bb25] Sheldrick, G. M. (2008). *Acta Cryst.* A**64**, 112–122. 10.1107/S0108767307043930 18156677

[bb26] Sheldrick, G. M. (2015). *Acta Cryst.* C**71**, 3–8.

[bb27] Spek, A. L. (2015). *Acta Cryst.* C**71**, 9–18. 10.1107/S2053229614024929 25567569

[bb28] Stapf, M., Seichter, W. & Mazik, M. (2015). *Chem. Eur. J.* **21**, 6350–6354. 10.1002/chem.201406383 25756753

[bb29] Stapf, M., Seichter, W. & Mazik, M. (2020*a*). *Acta Cryst.* E**76**, 1679–1683. 10.1107/S2056989020012554 PMC7534252 33117589

[bb30] Stapf, M., Seichter, W. & Mazik, M. (2020*b*). *Eur. J. Org. Chem.* pp. 4900–4915.

